# Intraspecific Variability of Wormwood (*Artemisia absinthium* L.) Occurring in Poland in Respect of Developmental and Chemical Traits

**DOI:** 10.3390/molecules30142915

**Published:** 2025-07-10

**Authors:** Olga Kosakowska, Zenon Węglarz, Agnieszka Żuchowska, Sylwia Styczyńska, Ewa Zaraś, Katarzyna Bączek

**Affiliations:** 1 Department of Vegetable and Medicinal Plants, Institute of Horticultural Sciences, Warsaw University of Life Sciences—SGGW, Nowoursynowska 166, 02-787 Warsaw, Poland; olga_kosakowska@sggw.edu.pl (O.K.); zenon_weglarz@sggw.edu.pl (Z.W.); agnieszka_zuchowska@sggw.edu.pl (A.Ż.); sylwia_styczynska@sggw.edu.pl (S.S.); 2 Department of Environmental Protection and Dendrology, Institute of Horticultural Sciences, Warsaw University of Life Sciences—SGGW, Nowoursynowska 166, 02-787 Warsaw, Poland; ewa_zaras@sggw.edu.pl

**Keywords:** wormwood, herbal raw materials, populations, essential oil, phenolics, chemotypes, Polish Genebank Collection

## Abstract

The aim of this study was to determine the intraspecific variability among 11 wild-growing populations of wormwood (*Artemisia absinthium* L.) originating from Central Europe and preserved in the Polish Genebank Collection. The populations were introduced into ex situ conditions, and assessed in terms of selected developmental and chemical traits (essential oil, phenolic acids, polyphenols, and tannins content). Developmental observations and harvest of raw materials were carried out in the second year of plant vegetation, at the beginning of flowering. The populations exhibited significant differences. The greatest variability was observed in the number of shoots per plant (38–51) and dry mass of herb per plant (0.83–1.60 kg). Essential oil (EO) content ranged from 0.75 to 1.69 g/100 g dry weight (DW). A total of 41 compounds were identified in the EOs, with oxygenated monoterpenes (such as sabinyl acetate, *cis*-chrysanthenol, chrysantenyl acetate, 1,8-cineole, α- and β-thujone) as dominants, showing considerable variation among populations. Based on the EO profiles, several chemotypes were distinguished, mainly (1) a pure sabinyl acetate chemotype; (2) mixed chemotypes with sabinyl acetate accompanied by β-myrcene, *cis*-chrysanthenol, chrysanthenyl acetate, or 1,8-cineole; and (3) a thujone chemotype. The total content of phenolic acids (expressed as caffeic acid equivalent), tannins, and polyphenols (as pyrogallol equivalent) varied significantly, too (0.37–0.50; 0.10–0.26; 0.58–0.79%, respectively). The results confirm a high level of intraspecific variability in both developmental and chemical traits of *A. absinthium* populations originating from Poland. This diversity may be valuable for future breeding programs and for the selection of populations with desired phytochemical profiles for medicinal, food, and agricultural applications. It is worth noting that the floristic diversity among populations indicates the habitat heterogeneity, ranging from natural or semi-natural (populations 1, 6) to more anthropogenically influenced ones (populations 2, 4, 5, 7–11).

## 1. Introduction

Plants belonging to the genus *Artemisia* (Asteraceae family) are widely distributed throughout the temperate zone of Europe and Asia. These perennials were also introduced to other parts of the world, especially North America and Australia. Several *Artemisia* species have been recognized as important medicinal plants, such as *A. annua*, *A*. *dracunculus*, *A. vulgaris*, and *A. absinthium* [1,2]. Among these, *Artemisia absinthium*, commonly known as wormwood, is the most widely investigated. Its aerial parts (herb; *Absinthi herba*), described in a European Pharmacopoeia (EP) monograph, are traditionally used to stimulate appetite and improve digestion [3,4]. The herb is particularly rich in EO, the content of which, according to EP requirements, should not be lower than 2 mL per kg [3]. The EO, chemically quite variable, is composed mainly of the following: α-thujone, β-thujone, β-pinene, sabinene, *cis*-epoxyocimene, sabinyl acetate, chrysanthenol, chrysanthenyl acetate, nerolidol, and chamazulene [5,6,7,8,9,10,11,12,13,14,15,16,17,18,19,20,21,22,23,24]. The raw material also contains significant amounts of non-volatile sesquiterpene derivatives, specifically sesquiterpene lactones of the guaianolide type (absinthin, anabsinthin, artabsinthin, matricin) and the pelenolide type (arabsin, artabin). Among these, absinthin, present at a level of 0.20–0.28%, is responsible for the herb’s characteristic bitter taste [4,10]. Other important constituents are phenolics, including phenolic acids, flavonoids, tannins, and coumarins. The most abundant phenolic acids are caffeoylquinic, dicaffeoylquinic, vanillic, coumaric, syringic, and chlorogenic acids. Flavonoids are mainly represented by artemetin, homorientin, quercetin, isorhamnetin, and their derivatives, while tannins are mainly represented by epigallocatechin gallate. Among coumarins, isofraxidine and scopolin have been identified here [1,4,25,26,27].

Wormwood exhibits digestive, choleretic, antispasmodic, carminative, hepatoprotective, and gastroprotective activity. The herb, considered as *aromatica amara*, is traditionally used to prepare tea infusions and alcoholic tinctures, applied mainly in the case of gastrointestinal disorders (flatulence, cramps) as a bitter aromatic medicine for temporary loss of appetite and to stimulate bile secretion in liver diseases [1,4,28]. Furthermore, wormwood herb exhibits antimicrobial, antioxidant, antipyretic, analgesic, anti-atherosclerotic, and anti-inflammatory activity [29,30,31,32,33,34,35,36,37,38,39,40]. It also demonstrates antiparasitic, especially anthelmintic, activity, resulting from the presence of α- and β-thujone [41,42]. However, due to the neurotoxic properties of these compounds, wormwood herb should be used only for a short period and in doses not exceeding the recommended limits. Toxicological studies of thujone are summarized, i.a., in the Public Statement on the use of herbal medicinal products containing thujone (EMA/HMPC/732886/2012) [43,44,45].

Given its broad spectrum of biological activity, the use of *Absinthi herba* is also recommended in veterinary practice, both internally, as a feed additive to prevent and treat gastrointestinal disorders, and externally against skin parasites such as lice and scabies [35,38,40,41,42]. Due to its strong antimicrobial activity, it is considered as a natural fungicide, effective mainly against *Fusarium*, *Alternaria*, and *Botrytis* strains [46,47]. Moreover, extracts from this plant have been shown to have insecticidal and repellent activity against *Sitophilus granarius* and *Rhyzopertha dominica* [48]. Due to its versatile applications, the plant has recently become the object of interest of various industries, especially the phytopharmaceutical, food, and agricultural sectors [1,2,28,29].

In Poland, wormwood herb is collected both from cultivation and, more commonly, from wild-growing plants [49,50]. The species grows naturally in various habitats, in a wide range of ecological conditions, mainly in grasslands, meadows, and ruderal and segetal communities. From a phytosociological perspective, wormwood is a characteristic species of the *Onopordetalia acanthii* order within the *Artemisietea vulgaris* class [51,52]. As mentioned earlier, the species is highly polymorphic. Raw materials collected from wild-growing plants are chemically heterogeneous, especially in terms of EO composition what may reduce its commercial quality. On the other hand, wild-growing populations can be a valuable source of genotypes useful for future breeding programs, particularly when searching for non-thujone forms [53].

The aim of the present work was to determine intraspecific variability of *Artemisia absinthium* growing wild in Poland in terms of developmental traits as well as the content and composition of biologically active compounds (essential oil, phenolic acids, polyphenols, and tannins) in the herb.

## 2. Results and Discussion

The phytosociological analysis indicates the heterogeneous character of wormwood plant communities (Table 1). Based on the species composition, two main types of communities may be distinguished here: ruderal/synanthropic communities, as well as sandy grasslands. In the frame of the first group, the presence of species such as *Oenothera biennis*, *Echium vulgare*, *Melilotus officinalis*, *Verbascum thapsus*, and *Artemisia absinthium* allow us to classify populations 2, 4, 5, 7, 8, 9, 10, and 11 as comprising the *Oenopordetalia acanthi* order within the *Artemisietea vulgaris* class. This type of plant community, characteristic for a wormwood occurrence, usually develops on sites disturbed by human activity [51,52]. In turn, populations 1 and 6 represent more natural or semi-natural dry habitats (sandy grasslands), with typical species such as *Thymus serpyllum*, *Helichrysum arenarium*, and *Sedum acre*. Populations 3 and 11 show species typical for meadow and ruderal communities, including nitrophilous plants such as *Urtica dioica* (Table 1).

The investigated populations varied in terms of developmental traits (Table 2). In general, the greatest variation was observed in the number of shoots per plant and internode length, whereas plant height and inflorescence length showed more uniformity across populations. The average plant height was at a level of 173.7 cm, with the highest plants observed in population 11 (196.0 ± 10.6 cm), which differed visibly from population 8 (165.2 ± 4.6 cm). Regarding the number of shoots per plant, a wide range (from 38 to 51) was noticed, followed by a high coefficient of variation (35.02%), indicating considerable variability within populations. Internode length varied from 21 mm in population 8 to 33 mm in population 1. Inflorescence length was relatively uniform across populations, with no significant differences observed. Here, the mean inflorescence length was 91.2 cm, with the longest recorded in populations 4 (112.7 ± 19.2 cm) and 11 (108.8 ± 13.6 cm) (Table 2). Such variability in morphological and developmental traits is typical for wild-growing plants, where phenotypic variation reflects their adaptation to the environment [49]. Moreover, features such as height, shoot number, and inflorescence length can be important from a practical viewpoint, since they affect the yield of herb and its mechanical harvest.

Both the mass of dry herb and grated herb exhibited relatively high variability within populations; however, these results were not statistically important (Table 3). The average dry herb mass per plant was at a level of 1.15 kg, with individual population values ranging from 0.83 kg (population 3) to 1.60 kg (population 6). Mass of grated herb, being a commercial raw material, was also variable: population 6 was characterized by the highest mass of grated herb (0.51 kg), and populations 1 and 2 by the lowest (0.26 kg) (Table 3).

Among examined wormwood populations, the content of EO varied from 0.94 to 1.69 g/100 g. Populations 7, 10, and 11 showed the highest content of this substance (1.47; 1.41; 1.69 g/100 g, respectively), visibly exceeding that in populations 1, 2, and 3 (0.94; 0.75 g/100 g) (Table 4). It is worth noting that according to EP requirements, EO content in wormwood herb should be not lower than 0.2 g/100 g [3]. Lachenmeier et al. (2006) claim that the amount of this substance ranges from 0.2% to 1.5% [45]. According to Juteau et al. (2003), the main factors influencing its accumulation are geographical origin of the population, stage of plant development, and the type of raw material. The authors noticed that the content of EO in wormwood herb decreases from the full blooming (0.68%) to the post-flowering stage (0.56%) of plant vegetation. Moreover, the upper part of flowering shoots contains 2–3 more EOs in comparison to lower parts [8].

In the present work, 41 compounds were detected in the EOs, comprising 81.02% to 97.18% of the whole fraction, depending on the population (Table 5). Identified compounds were classified as monoterpenes and sesquiterpenes, both in the form of hydrocarbons and oxygenated derivatives, as well as other group such as esters, alcohols, and aldehydes, present in minor concentrations. The oxygenated monoterpenes were a fundamental part, accounting for 41.10% (population 4) to 90.34% (population 8) of the total EOs. The main oxygenated monoterpenes included 1,8-cineole, sabinyl acetate, *cis*-chrysanthenol, and chrysantenyl acetate as well as α- and β-thujone. Their content exhibited remarkable variations depending on the population. The content of 1,8-cineole ranged from 0.47 to 40.64%, and was predominant in both populations 3 (40.64%) and 5 (28.93%). Sabinyl acetate was found as highly abundant in populations 11 (76.02%), 7 (56.80%), 8 (41.91%), 6 (39.02%), 10 (30.77%), and 1 (17.37%), being nearly absent in the others. *Cis*-chrysanthenol was present in significant amount in populations 1 (32.09%) and 2 (49.48%) while its derivative chrysanthenol acetate was present only in population 8 (19.83%). Regarding the two isomers of thujone, α-thujone and β-thujone, both were present in remarkable amounts only in population 9 (19.31% and 19.26%, respectively). Monoterpene hydrocarbons varied among populations, ranging from 1.26% in population 8 to 44.04% in population 4. Major compounds in this group included β-myrcene, with the highest concentrations recorded in populations 4 (25.76%) and 5 (16.33%). Sesquiterpene hydrocarbons and oxygenated sesquiterpenes were present in smaller quantities, usually below 4%, with slight variability between populations. Among the more significant compounds in this group were caryophyllene, α-humulene, and β-copaene (Table 5).

**Figure 1:**
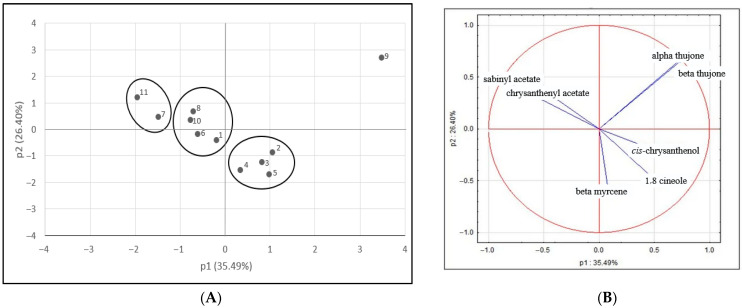
PCA analysis: (**A**) principal components analysis (PCA) performed on the major EO component for the analysed populations; (**B**) grouping of phytochemical compounds obtained from essential oils according to their first and second components.

Taking into account such a variable composition of the EOs, several chemotypes may be distinguished within the examined wormwood populations. Here, populations 11 and 7 may be qualified as a pure sabinyl acetate chemotype. Populations 1, 6, 8, and 10 constitute mixed chemotypes where sabinyl acetate is accompanied by β-myrcene, *cis*-chrysanthenol, chrysanthenyl acetete, and 1,8-cineole. Population 9 alone represents a mixed chemotype dominated by thujones followed by *cis*-chrysanthenol. Finally, populations 2, 3, 4, and 5 represent mixed chemotypes with β-myrcene, 1,8-cineole, *cis*-chrysanthenol, and *cis*-epoxyocymene as dominants (Figure 1).

The obtained results are in good agreement with the literature data and confirm the highly variable character of wormwood. The chemical diversity of wormwood essential oil is well established, with numerous studies confirming that both genetic and environmental factors, as well as plant developmental stage and organ specificity, contribute to its pronounced polymorphism [5,6,7,8,9,10,11,12,13,14,15,16,17,18,19,20,21,22,23,24].

Globally, α-thujone and β-thujone are often regarded as the most frequent dominant compounds in wormwood EO, yet this is only one aspect of a highly diverse species. In Central Europe, at least four main chemotypes have been identified: α- and β-thujone, sabinene and β-myrcene, *cis*-epoxyocimene, and sabinyl acetate [11,12,26]. Regional differences are worth noting; for example, in Lithuania, thujones and *trans*-sabinyl acetate predominate, whereas in Poland, sabinyl acetate, chrysanthenyl acetate, and sabinene chemotypes are most common, with β-thujone-dominant types occurring only rarely [11,26]. In France, EO profiles can be dominated by *cis*-chrysanthenol or *cis*-epoxyocimene, while in the Spanish Pyrenees, *cis*-epoxyocimene (up to 49.7%) or its combination with *cis*-chrysanthenyl acetate (up to 36.7%) is typical. Italian and Spanish populations may also contain significant levels of 1,8-cineole (18.0%), carvone (18.5%), thymol (10.8%), and carvacrol (9.7%) [13,14]. Recent comprehensive analyses confirm that the chemotype spectrum in *A. absinthium* is even broader. For instance, Raal et al. (2024) examined commercial EO samples from 14 European countries, identifying 41 compounds and revealing that while monoterpenes and their derivatives dominate, the quantitative composition varies widely. Pure chemotypes such as E-sabinyl acetate and E-epoxyocymene were observed, as well as numerous mixed chemotypes, with major constituents differing even within a single country. This variability is not strictly associated with geographic or altitudinal gradients, indicating a complex interplay of factors shaping EO profiles [15].

Outside Europe, Egyptian wormwood is characterized by high α-phellandrene and terpinen-4-ol content, and Iranian populations may be rich in β-pinene [16,17]. A particularly instructive example of environmental impact comes from Tunisia, where Riahi et al. (2015) investigated *A. absinthium* from four bioclimatic zones ranging from humid to arid. They found high qualitative and quantitative variation in EO composition depending on locality. For example, plants from the Inferior Arid region (Gafsa) had chamazulene, α-thujone, and camphor as main constituents, while those from the humid zone (Ghar Dimaou) were characterized by high camphor, Z-sabinene hydrate, and 1-terpinen-4-ol. The antioxidant activity of the essential oils also increased significantly from humid to arid zones, correlating with higher thujone and chamazulene content in arid environments. These results highlight that both environmental and genetic factors shape the chemotype and biological activity of wormwood EO, and that adaptation to local stressors (such as drought) may drive the synthesis of specific bioactive compounds [18].

Environmental conditions and developmental stages significantly influence EO composition as well. Malaspina et al. (2025) demonstrated that both geographical location and phenological stage (vegetative versus flowering) can shift the dominant chemical classes in the essential oil. For example, in Northern Italy, oxygenated monoterpenes prevailed, while in Southern Italy, oxygenated sesquiterpenes or monoterpene hydrocarbons dominated depending on the developmental stage. The main EO constituents also changed: myroxide and 3,6-dihydrochamazulene were abundant in Cogne, whereas camphor, davanone, β-myrcene, and β-phellandrene were prominent in Ogliastro [19].

Organ- and stage-specific differences are also significant. Nguyen et al. (2018) showed that EO content is generally higher in flowers than in leaves, and the proportions of key compounds such as thujones and sabinyl acetate change during plant development. In thujone chemotypes, α- and β-thujone are most abundant at early stages and decrease after flowering, while in *trans*-sabinyl acetate chemotypes, this ester declines over time with a concomitant increase in thujones post flowering. These changes are primarily quantitative, but qualitative differences between organs and stages were also observed [20].

Investigated populations were differentiated by the content of phenolic compounds, including polyphenols, tannins, and phenolic acids (Table 4). Average polyphenols content reached 0.66 g/100 g (CV = 11.77%), with populations 5 and 6 characterized by their highest level (0.79 g/100 g), and populations 2, 9, and 11 characterized by the lowest (around 0.58–0.60 g/100 g). The content of polyphenols in wormwood was investigated earlier by other authors [25,26,28]. In our work, phenolic acids content was at a level of 0.42 g/100 g with moderate variability (CV = 13.04%), with populations 1 and 9 distinguished by the highest amount of these compounds (0.50 g/100 g). Tannin content was more variable (CV = 28.04%), ranging from 0.10 g/100 g in population 2 to 0.26 g/100 g in population 5 (Table 4).

In general, such chemical variability within the content of biologically active compounds is often driven by genetic differences and environmental influences, which affect their biosynthesis and accumulation in plant tissues. From a physiological perspective, both EOs and phenolics play crucial roles in plant stress responses [54,55,56]. These compounds contribute to antioxidative protection by scavenging reactive oxygen species generated under abiotic stresses like drought, UV radiation, and temperature extremes, as well as biotic stresses such as pathogen attack. Tannins and EO, in particular, may inhibit microbial (including bacteria and fungi) growth, enhancing plant survival in wild habitats [54,55,56,57,58,59]. The reported coefficients of variation indicate that both tannin and EO content in the examined populations is particularly variable (CV = 28.04 and 23.89%, respectively), suggesting that these classes of compounds are responsive to environmental factors. In turn, polyphenols and phenolic acids showed moderate but statistically significant variation (CV = 11.77% and 13.04%, respectively).

The observed variability provides opportunities for selecting wormwood populations with desirable traits for cultivation and commercial use, both medicinal and non-medicinal. The medicinal properties of the abovementioned phenolic compounds present in wormwood herb are well-documented. They are considered mainly as strong antioxidants, exhibiting also antimicrobial, anti-inflammatory, and astringent activities [25,26,27,28,29,30,31]. Therefore, populations 5 and 6 with an elevated content of polyphenols and tannins appeared to be valuable from a medicinal viewpoint. Given the EO content, all populations meet or even exceed European Pharmacopoeia standards [3]. Due to the biological activity of sabinyl acetate, both pure and mixed chemotypes with this as the dominant compound may contribute to medicinal, e.g., antimicrobial and anti-inflammatory, applications [60,61]. However, the use of the thujone-rich chemotype (population 9), due to its neurotoxic activity, would be limited for direct human consumption. Nevertheless, thujone’s strong repellent activity against pests and bactericidal effects on phytopathogens make it valuable biopesticide in organic crop protection [62,63,64].

To sum up, our results indicate that population 6 stands out as the most promising candidate for practical applications. This population achieved the highest average dry herb mass per plant (1.60 kg) and the highest mass of grated herb (0.51 kg). The essential oil content in this population, reaching 1.21 g/100 g, exceeds the minimum requirements set by the European Pharmacopoeia. From a quality perspective, population 6 represents a mixed sabinyl acetate chemotype, characterized by a high proportion of sabinyl acetate (39.02%) and a favorable profile of oxygenated monoterpenes, while maintaining a low content of thujones. Additionally, population 6 is distinguished by a high content of polyphenols (0.79 g/100 g) and an elevated tannin level (0.22 g/100 g), both of which are associated with antioxidant and antimicrobial activities, enhancing the therapeutic and functional value of the raw material. All these makes population 6 particularly attractive for cultivation and further breeding.

## 3. Materials and Methods

**Figure 2:**
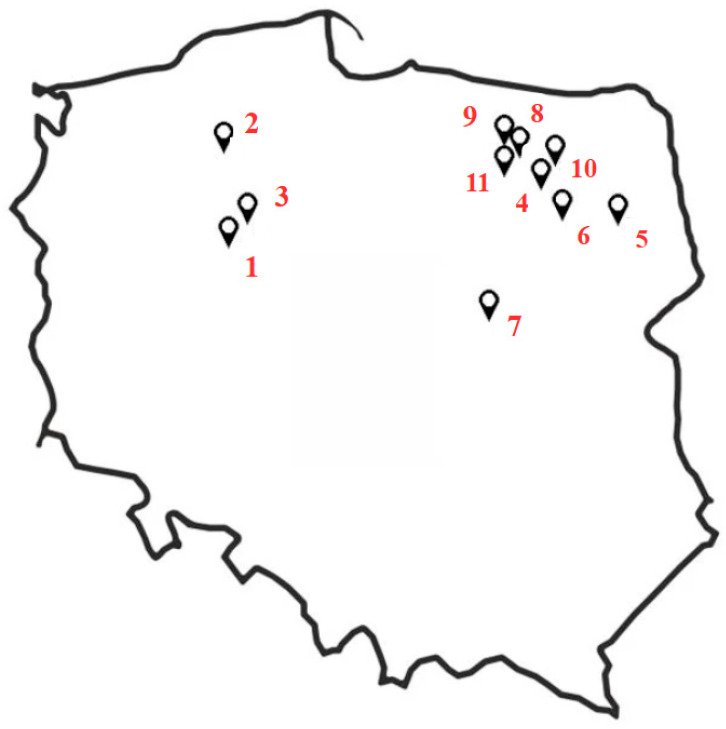
The map of Poland with the examined natural sites of wormwood (origin of population 1–11).

The objects of the study were 11 wormwood populations originating from northern-east and central Poland, introduced to ex situ conditions (Table 6, Figure 2). At each natural site the basic floristic observations were carried out [51,52].

### 3.1. Field Experiment

In 2023, at each identified natural site, the seedlings of wormwood (young plants in the vegetative stage) were collected in order to establish a field experiment. The research was conducted in an experimental field of Department of Vegetable and Medicinal Plants, WULS-SGGW, on alluvial soil. On a single plot, 15 seedlings were planted out, with a spacing of 50 × 80 cm. The developmental observations and harvest of raw material was carried out in the second year of plant vegetation (2024), at the beginning of blooming (July). The collected herb was dried at 35 °C and grated herb was subjected to chemical analysis. Soil characteristics and climatic parameters of the 2024 vegetation season were recorded (Table 7 and Table 8). During the vegetation season, irrigation was applied when required to maintain optimal soil moisture.

The voucher specimens of the populations’ seeds collected in natural habitats are kept in the National Centre for Plant Genetic Resources (Polish GeneBank), while representative plants are kept in the living collection of medicinal and aromatic plants of the Department of Vegetable and Medicinal Plants.

### 3.2. Developmental Characteristics

Directly before the harvest of raw materials, the following measurements of plants were performed: plant height (cm), number of shoots per plant, length of the internode (mm), and length of inflorescence (cm). Dry mass of herb and mass of grated herb (kg/plant) were determined, as well. At each population, the observations were conducted on 15 plants.

### 3.3. Chemical Analysis

#### 3.3.1. Essential Oil Content

A total of 30 g of air-dried raw material was obtained by 2 h hydro-distillation using the Clevenger-type apparatus (Chemland, Warsaw, Poland). The content of EO was expressed as g/100 g of dry weight (DW). Essential oils were collected and stored in amber vials, at 4 °C.

#### 3.3.2. Analysis of Essential Oils by GC/MS

The qualitative GC-MS analysis was carried out using Shimadzu GC2050 (Kyoto, Japan) equipped with autosampler AOC30i (Shimadzu, Kyoto, Japan) and polar column SH-PolarWax-MS (Shimadzu, Kyoto, Japan) (30 m; 0.25 mm; 0.25 µm). The operating conditions were as follows: oven temperature 2 min. isothermal at 60 °C, then rising at 4 °C per min to 210 °C and held isothermal for 5 min. Injector temperature: 210 °C. The carrier gas (He) flow was 1.1 mL·min^−1^. The split ratio was 1:20. Diluted samples (5/1000 *v*/*v*, in n-hexane) of 1 μL were injected at 210 °C by auto sampler. Ion source temperature −220 °C, ionization voltage 70 eV. Mass spectra were scanned in the range 40–500 amu. Essential oil compounds identification was based on comparison of mass spectra from the Mass Spectral Database, i.e., NIST20R and FNSSC, and on comparison of retention indices (RI) relative to retention times of a series of n-hydrocarbons (C7–C30) with those reported in literature [65].

#### 3.3.3. Total Content of Phenolic Acids

The analyses were carried out according to Polish Pharmacopeia 6th ed. [66]. Measurement of phenolic acids content was carried out by Arnov’s method. A total of 1 g of air-dried, powdered raw material was extracted twice with portions of 25 mL of distilled water (a total of 50 mL), with shaking at room temperature for 30 min each time (a total of 1 h). Cumulated extract was completed to 50 mL with distilled water. A total of 1 mL of extract was mixed with 5 mL of distilled water, 1 mL 0.5 M HCl, 1 mL of Arnov reagent (10 g of sodium molybdate and 10 g of sodium nitrite dissolved in 100 mL of distilled water), and 1 mL 1 M NaOH and subsequently diluted to 10 mL with distilled water. The absorbance was measured at 490 nm for both basic (with extract) and comparison (without extract) solutions. The total phenolic acid content was expressed as caffeic acid equivalent (g/100 g DW).

#### 3.3.4. Total Content of Tannins and Polyphenols

The analysis was carried out according to Polish Pharmacopoeia 6th ed. [66]. Total tannin content was determined using the Folin–Ciocalteu method. A total of 1 g of air-dried, powdered raw material was extracted with 150 mL of distilled water for 30 min. The mixture was cooled, transferred to a 250 mL volumetric flask, and made up to volume with water. After sedimentation, the extract was filtered, discarding the first 50 mL. For the determination of total polyphenols, 5 mL of filtrate was diluted to 25 mL with water. A total of 2 mL of this solution was mixed with 1 mL Folin–Ciocalteu reagent, 10 mL water, and completed to 25 mL with sodium carbonate solution (290 g/L). Absorbance (A_1_) was measured at 760 nm after 30 min. For non-tannin polyphenols, 10 mL of filtrate was shaken with 0.1 g hide powder for 1 h, filtered, and 5 mL was diluted to 25 mL with water. A total of 2 mL of this solution was treated as above and absorbance (A_2_) was measured. For the standard, 50 mg pyrogallol was dissolved in 100 mL water, 5 mL was diluted to 100 mL, and 2 mL was treated as above and absorbance (A_3_) measured. After recalculation, the results were expressed as pyrogallol equivalents (g/100 g DW).

### 3.4. Statistical Analysis

Data were subjected to a statistical analysis using the Statistica 12 software (Krakow, Poland). All observations and analysis were performed min. in triplicate. The mean values were compared using the one-way analysis of variance (ANOVA) and expressed as mean with standard deviation (±SD). The differences between individual means were signed as different letters and considered to be significant at *p* < 0.05. Principal component analysis (PCA) was performed to identify the main components of the essential oil and to differentiate amongst population groups according to their chemical similarity.

## Figures and Tables

**Table 1:** Other plant species present on the investigated natural sites.

Population No.	Plant Species
1	*Achillea millefolium* L., *Vicia cracca* L., *Plantago lanceolata* L., *Prunus spinosa* L., *Helichrysum arenarium* (L.) *Moench*, *Echium vulgare* L., *Malus sylvestris* Mill., *Oenothera biennis* L., *Pinus sylvestris* L., *Thymus serpyllum* L., *Galium mollugo* L., *Artemisia absinthium* L.
2	*Conyza canadensis* (L.), *Artemisia campestris* L., *Daucus carota* L., *Cichorium intybus* L., *Artemisia vulgaris* L., *Senecio jacobaea* L., *Silene vulgaris* (Moench) Garcke, *Achillea millefolium* L., *Tripleurospermum inodora* (L.) Sch.Bip., *Taraxacum officinale* F.H.Wigg., *Capsella bursa-pastoris* (L.) Medik., *Eryngium campestre* L., *Cirsium* sp., *Artemisia absinthium* L.
3	*Potentilla anserina* L., *Taraxacum officinale* F.H.Wigg., *Plantago major* L., *Urtica dioica* L., *Galium verum* L., *Artemisia absinthium* L.
4	*Plantago major* L., *Polygonum aviculare* L., *Trifolium pratense* L., *Daucus carota* L., *Artemisia campestris* L., *Medicago lupulina* L., *Artemisia absinthium* L.
5	*Taraxacum officinale* F.H.Wigg., *Echium vulgare* L., *Achillea millefolium* L., *Trifolium pratense* L., *Plantago major* L., *Polygonum aviculare* L., *Sambucus nigra* L., *Equisetum arvense* L., *Crepis biennis* L., *Melilotus officinalis* (L.) Pall., *Artemisia absinthium* L.
6	*Hypericum perforatum* L., *Echium vulgare* L., *Plantago major* L., *Polygonum aviculare* L., *Urtica dioica* L., *Thymus serpyllum* L., *Betula pendula* Roth., *Euphorbia cyparissias* L., *Sedum acre* L., *Senecio sylvaticus* L.
7	*Pinus sylvestris* L., *Chelidonium majus* L., *Daucus carota* L., *Prunus spinosa* L., *Tanacetum vulgare* L., *Cichorium intybus* L., *Symphytum officinale* L., *Artemisia absinthium* L.
8	*Cichorium intybus* L., *Echium vulgare* L., *Achillea millefolium* L., *Tussilago farfara* L., *Equisetum arvense* L., *Melilotus officinalis* (L.) Pall., *Prunus spinosa* L., *Betula pendula* Roth, *Artemisia absinthium* L.
9	*Artemisia vulgaris* L., *Plantago major* L., *Achillea millefolium* L., *Rumex acetosella* L., *Chelidonium majus* L., *Tanacetum vulgare* L., *Malva neglecta* Wallr., *Polygonum aviculare* L., *Taraxacum officinale* F.H.Wigg., *Trifolium pratense* L., *Galium mollugo* L., *Galium verum* L. *Artemisia absinthium* L.
10	*Potentilla anserina* L., *Plantago major* L., *Achillea millefolium* L., *Chelidonium majus* L., *Echium vulgare* L., *Tanacetum vulgare* L., *Malva neglecta* Wallr., *Polygonum aviculare* L., *Taraxacum officinale* F.H.Wigg., *Oenothera biennis* L., *Artemisia absinthium* L.
11	*Taraxacum officinale* F.H.Wigg., *Achillea millefolium* L., *Equisetum arvense* L., *Plantago lanceolata* L., *Potentilla anserina* L., *Galium verum* L., *Artemisia absinthium* L.

**Table 2:** Developmental traits of investigated populations.

Population	Plant Height (cm)	Number of Shoots per Plant	Length of Internodes (mm)	Length of Inflorescences (cm)
1	165.8 ± 15.6 ab	38 ± 10	33 ± 4 b	92.1 ± 3.2
2	177.4 ± 12.0 ab	51 ± 12	27 ± 1 ab	90.5 ± 11.1
3	165.6 ± 10.9 ab	46 ± 12	27 ± 4 ab	82.3 ± 2.1
4	160.2 ± 15.3 a	43 ± 20	25 ± 1 ab	112.7 ± 19.2
5	176.4 ± 15.1 ab	39 ± 6	28 ± 0 ab	103.2 ± 12.5
6	172.8 ± 9.3 ab	42 ± 11	23 ± 2 ab	74.2 ± 10.5
7	186.6 ± 16.7 ab	41 ± 18	24 ± 4 ab	81.4 ± 7.6
8	165.2 ± 4.6 a	44 ± 15	21 ± 2 a	79.3 ± 12.7
9	166.6 ± 9.5 ab	44 ± 14	24 ± 3 ab	88.5 ± 12.3
10	177.6 ± 15.9 ab	48 ± 15	26 ± 1 ab	90.4 ± 12.3
11	196.0 ± 10.6 b	39 ± 18	24 ± 2 ab	108.8 ± 13.6
mean	173.7	43.5	26	91.2
CV	9.42	35.02	16.25	18.13

α = 0.05; a, b—homologues groups.

**Table 3:** Mass of herb (kg/plant).

Population	Dry Herb	Dry Grated Herb
1	0.87 ± 0.19	0.26 ± 0.07
2	1.30 ± 0.32	0.26 ± 0.08
3	0.83 ± 0.30	0.28 ± 0.06
4	1.20 ± 0.27	0.41 ± 0.12
5	1.50 ± 0.18	0.42 ± 0.15
6	1.60 ± 0.29	0.51 ± 0.15
7	0.98 ± 0.28	0.29 ± 0.11
8	0.92 ± 0.36	0.48 ± 0.14
9	1.12 ± 0.59	0.33 ± 0.12
10	1.25 ± 0.11	0.37 ± 0.11
11	1.13 ± 0.33	0.34 ± 0.09
mean	1.15	0.36
CV	34.17	24.00

α = 0.05.

**Table 4:** The total content of biologically active compounds (g/100 g).

Population	Essential Oil	Phenolic Compounds		
		Polyphenols	Tannins	Phenolic Acids
1	0.94 ± 0.04 ab	0.72 ± 0.03 bc	0.24 ± 0.06 cd	0.50 ± 0.05 c
2	0.94 ± 0.20 ab	0.59 ± 0.04 a	0.10 ± 0.01 a	0.39 ± 0.05 ab
3	0.75 ± 0.03 a	0.66 ± 0.06 ab	0.20 ± 0.01 bcd	0.39 ± 0.04 ab
4	0.99 ± 0.02 abc	0.66 ± 0.03 ab	0.14 ± 0.03 ab	0.45 ± 0.03 abc
5	1.11 ± 0.06 bc	0.79 ± 0.03 c	0.26 ± 0.03 d	0.43 ± 0.05 abc
6	1.21 ± 0.06 cd	0.79 ± 0.01 c	0.22 ± 0.02 bcd	0.46 ± 0.01 abc
7	1.47 ± 0.06 de	0.63 ± 0.01 ab	0.21 ± 0.02 bcd	0.37 ± 0.02 a
8	1.23 ± 0.05 cd	0.67 ± 0.05 ab	0.23 ± 0.04 bcd	0.39 ± 0.02 ab
9	1.09 ± 0.04 bc	0.60 ± 0.01 a	0.14 ± 0.01 ab	0.48 ± 0.04 bc
10	1.41 ± 0.06 d	0.61 ± 0.01 ab	0.21 ± 0.01 bcd	0.40 ± 0.03 abc
11	1.69 ± 0.07 e	0.58 ± 0.01 a	0.16 ± 0.01 abc	0.39 ± 0.03 ab
mean	1.18	0.66	0.19	0.42
CV	23.89	11.77	28.04	13.04

α = 0.05, a–e—homologues groups.

**Table 5:** The chemical composition of EOs (%).

	Compound		Populations										
		RI	1	2	3	4	5	6	7	8	9	10	11
**1**	β-myrcene	1160	12.39	3.33	4.19	25.76	16.33	10.19	12.02	0.77	3.53	4.00	1.59
**2**	d-limonene	1204	0.07	0.15	0.32	0.24	0.15	nd	0.07	0.10	0.14	0.12	0.12
**3**	1,8-cineole	1214	7.12	19.71	40.64	18.58	28.93	14.87	0.47	13.06	16.18	6.77	2.18
**4**	γ-terpinene	1238	0.13	0.26	1.45	0.48	nd	0.29	0.19	0.22	0.10	0.33	0.16
**5**	*cis*-epoxyocymene	1296	0.53	0.28	0.21	17.56	1.19	0.37	nd	0.17	1.22	0.12	0.10
**6**	hex-(3*Z*)-enol	1372	0.10	nd	0.10	nd	0.20	nd	0.13	nd	0.10	0.11	0.08
**7**	nonanal	1391	0.13	0.19	nd	0.14	nd	nd	nd	0.10	0.11	nd	0.06
**8**	α-thujone	1425	0.16	0.11	0.09	1.01	nd	0.19	0.95	0.23	19.31	0.16	0.14
**9**	1-octen-3-ol	1446	0.27	1.24	0.64	0.37	0.19	0.42	0.42	0.22	0.18	0.23	0.06
**10**	β-thujone	1448	0.09	0.23	0.12	1.13	0.65	0.74	0.06	1.12	19.26	0.12	0.13
**11**	sabinene hydrate	1451	0.14	0.10	1.93	0.16	0.33	0.33	0.20	0.15	nd	0.19	0.21
**12**	sabinyl acetate	1464	17.37	0.91	nd	0.80	0.71	39.02	56.80	41.91	0.31	30.77	76.02
**13**	linalool	1542	2.41	2.28	4.02	4.01	3.58	2.91	1.21	2.58	2.53	6.90	0.48
**14**	chrysanthenyl acetate	1560	6.16	0.14	nd	nd	0.16	nd	nd	19.83	0.15	4.37	0.13
**15**	β-copaene	1580	1.63	nd	nd	nd	0.51	0.57	0.39	1.25	nd	nd	0.16
**16**	linalyl acetate	1581	0.24	nd	0.11	nd	0.15	nd	nd	nd	nd	nd	nd
**17**	caryophyllene	1594	1.82	3.30	3.15	3.21	1.90	1.37	2.28	0.69	nd	1.22	1.80
**18**	terpinen-4-ol	1597	1.53	1.01	3.45	4.76	4.55	0.84	0.54	1.08	1.21	1.90	0.51
**19**	α-humulene	1658	0.22	0.31	nd	0.42	0.16	0.12	0.22	nd	0.15	0.13	0.15
**20**	δ-terpineol	1662	0.17	0.28	nd	0.33	0.35	0.16	nd	0.43	0.19	nd	nd
**21**	isoborneol	1670	0.11	0.19	nd	nd	nd	nd	nd	nd	0.14	0.47	nd
**22**	lavandulol	1675	2.60	0.67	nd	0.99	0.14	nd	0.49	0.17	0.27	nd	0.11
**23**	lavandulyl isobutyrate	1677	0.90	1.19	1.66	1.91	1.61	1.27	0.49	1.14	1.03	2.16	0.50
**24**	lavandulyl isovalerate	1680	0.81	0.42	nd	0.47	nd	nd	0.08	nd	nd	nd	nd
**25**	neral	1688	0.08	nd	0.18	0.22	0.12	nd	nd	0.09	nd	nd	nd
**26**	α-terpineol	1694	1.15	2.86	6.10	3.16	4.41	2.26	0.17	2.15	2.55	1.14	0.59
**27**	*trans*-sabinol	1717	1.25	0.25	0.21	0.20	nd	6.21	8.79	3.07	0.15	2.96	2.87
**28**	geranial	1725	0.07	nd	nd	0.58	0.14	nd	nd	nd	nd	nd	0.64
**29**	neryl isobutyrate	1761	0.52	0.60	1.32	0.55	0.88	0.76	0.27	0.53	0.53	0.68	0.25
**30**	*cis*-chrysanthenol	1766	32.09	49.48	6.20	nd	26.16	6.51	0.20	nd	21.16	15.89	0.58
**31**	nerol	1770	0.65	0.35	1.14	0.69	0.92	0.45	0.25	0.46	0.71	nd	0.34
**32**	geranyl acetate	1780	0.10	0.15	0.13	0.19	0.43	0.25	nd	0.26	0.15	0.22	0.06
**33**	geranyl isobutanoate	1790	0.20	1.36	0.20	nd	nd	1.45	1.25	1.14	0.14	2.22	1.01
**34**	tridecanol	1818	0.19	0.43	nd	nd	0.40	0.14	nd	nd	0.21	0.09	nd
**35**	ionone	1838	0.39	0.15	0.45	0.23	nd	nd	0.31	nd	nd	0.17	nd
**36**	hexanoic acid	1843	0.12	0.38	nd	nd	0.20	nd	nd	0.26	0.71	0.33	nd
**37**	geraniol	1845	0.12	0.09	0.32	0.15	0.15	0.22	0.07	0.17	0.15	0.13	0.17
**38**	neryl isovalerate	1870	0.60	0.50	1.33	1.03	1.07	1.12	0.35	0.77	0.77	1.13	0.47
**39**	geranyl isovalerate	1904	0.33	0.45	nd	nd	nd	0.16	0.27	nd	0.14	0.18	0.19
**40**	jasmone <(E)->	1955	0.19	0.21	0.23	0.18	0.31	nd	0.07	nd	nd	0.13	nd
**41**	14-hydroxycaryophyllene	2350	0.72	0.73	1.13	1.87	0.20	0.53	0.94	0.33	nd	0.41	0.29
	Monoterpene hydrocarbons		13.12	4.02	6.17	44.04	17.67	10.85	12.28	1.26	4.99	4.57	1.97
	Oxygenated monoterpenes		76.92	83.54	69.27	41.10	75.60	79.72	72.98	90.34	87.03	78.49	87.58
	Sesquiterpene hydrocarbons		3.91	3.61	3.26	3.63	2.72	2.06	2.89	1.94	0.15	1.35	2.11
	Oxygenated sesquiterpenes		0.72	0.73	1.13	1.87	0.20	0.53	0.94	0.33	nd	0.41	0.29
	Other		1.20	2.39	1.19	0.74	0.99	0.56	0.86	0.58	1.31	0.93	0.20
	Total		95.87	94.29	81.02	91.38	97.18	93.72	89.95	94.45	93.48	85.75	92.15

nd—not detected.

**Table 6:** Geographical location of natural sites of wormwood populations.

Population No	Accesion No	Coordinates		Voivodeship	Altitude
1	407,479	53°43′ N	21°47′ E	kujawsko-pomorskie	71
2	407,480	53°48′ N	21°39′ E	pomorskie	202
3	407,481	53°39′ N	22°23′ E	kujawsko-pomorskie	69
4	407,482	53°36′ N	21°35′ E	podlaskie	183
5	407,483	53°23′ N	22°15′ E	podlaskie	135
6	407,485	53°07′ N	23°15′ E	podlaskie	99
7	407,484	52°18′ N	21°15′ E	mazowieckie	105
8	407,587	53°12′ N	22°26′ E	warmińsko-mazurskie	104
9	407,586	53°03′ N	17°26′ E	warmińsko-mazurskie	108
10	407,588	53°51′ N	17°20′ E	warmińsko-mazurskie	95
11	407,589	53°06′ N	17°30′ E	warmińsko-mazurskie	159

**Table 7:** Soil parameters (pH, the content of main nutrients (mg·L^−1^), and salinity (g KCl·L^−1^).

pH	NO_3_^−^	NH_4_^+^	P	K	Ca	Mg	Cl	Salinity
5.83	61	8	19	50	739	95	<20	0.55

**Table 8:** Climatic parameters in the vegetation season of 2024.

Months	Temperature (°C)	Rainfall (mm)	Sun Hours	Sun Days
April	11	39.79	161	85
May	17			
June	20			
July	22	31.65	227	88
August	23			
September	20			

## Data Availability

The original contributions presented in this study are included in the article/Supplementary Material. Further inquiries can be directed to the corresponding author.

## Supplementary Materials

The following supporting information can be downloaded at: https://www.mdpi.com/article/10.3390/molecules30142915/s1, Figure S1. Natural site of population 1; Figure S2. Natural site of population 2; Figure S3. Natural site of population 3; Figure S4. Natural site of population 5; Figure S5. Natural site of population 7; Figure S6. Natural site of population 11; Figure S7. Young plants of wormwood in the vegetative stage (seedling); Figure S8. Seedlings preparation; Figure S9. Well-rooted seedlings; Figure S10. Plantation establishment; Figure S11. Plants in the first year of the vegetation; Figure S12. Plants in the second year of the vegetation; Figure S13. EO chromatogram of population 4; Figure S14. EO chromatogram of population 9; Figure S15. EO chromatogram of population 10; Table S1. One-way Analysis of Variance parameters (DF—the degrees of freedom; SS—the sum of squares; MS—the mean sum of squares; F—the F-statistic; P—the *p*-value).
