# Supplementary material for: Intraspecific Variability of Wormwood (Artemisia absinthium L.) Occurring in Poland in Respect of Developmental and Chemical Traits
Source: Molecules. 2025 Jul 10;30(14):2915. doi: 10.3390/molecules30142915 (PMC12299115; doi:10.3390/molecules30142915)
Supplement: Supplementary file 1 [file molecules-30-02915-s001.zip › molecules-3729852-supplementary.pdf]

Supplementary materials

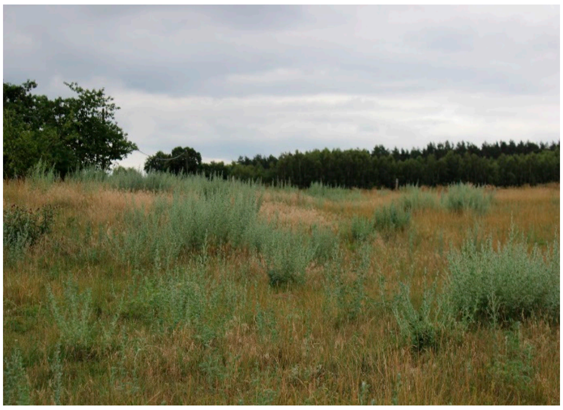

Figure S1. Natural site of population 1

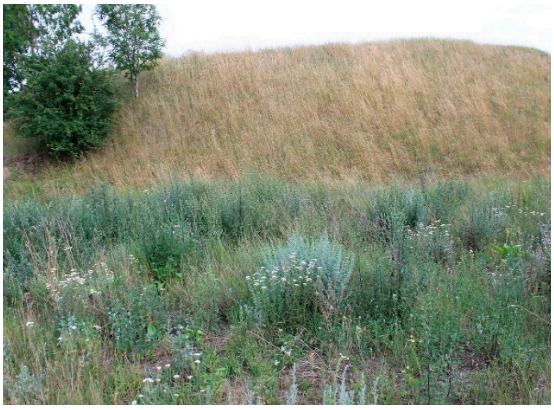

Figure S2. Natural site of population 2

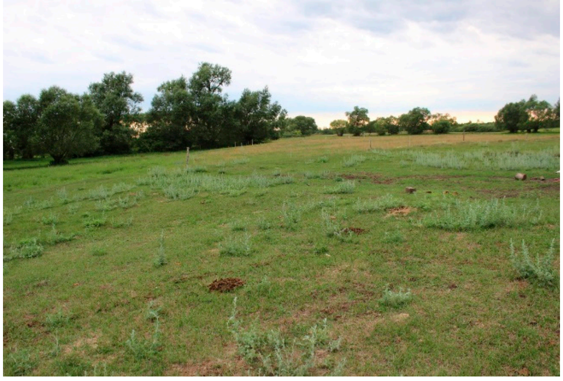

Figure S3. Natural site of population 3

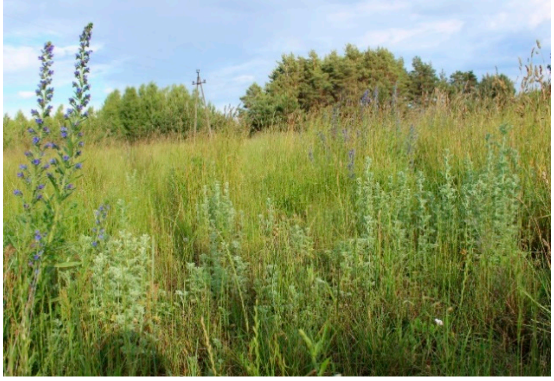

Figure S4. Natural site of population 5

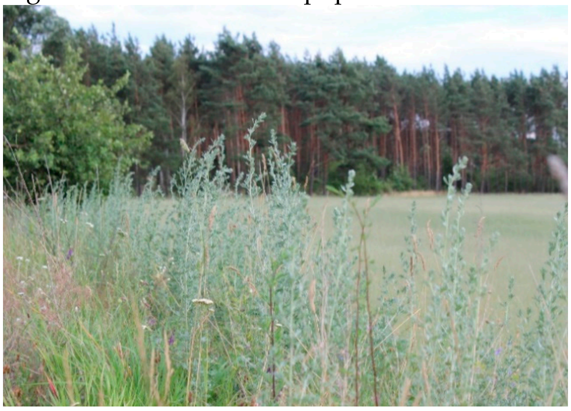

Figure S5. Natural site of population 7

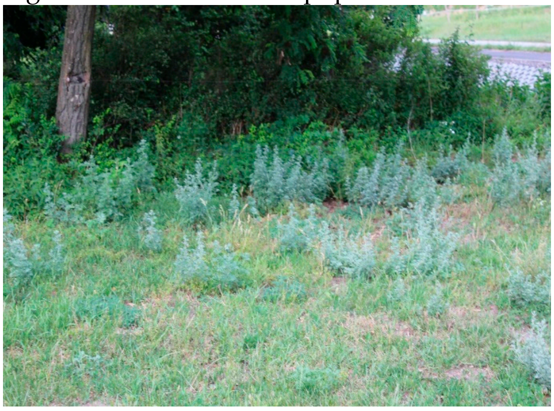

Figure S6. Natural site of population 11

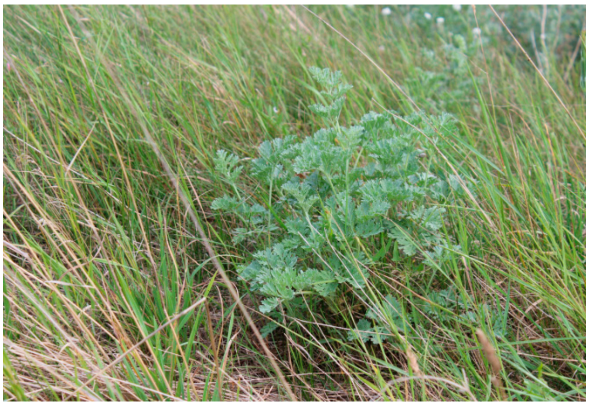

Figure S7. Young plants of wormwood in the vegetative stage (seedling)

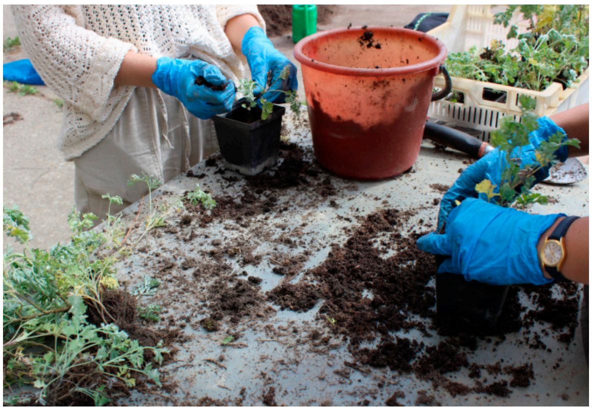

Figure S8. Seedlings preparation

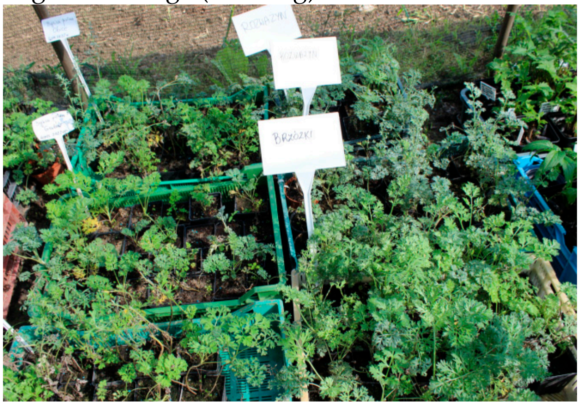

Figure S9. Well-rooted seedlings

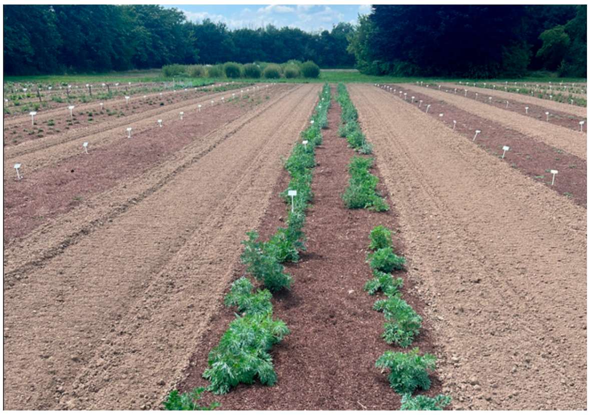

Figure S10. Plantation establishment

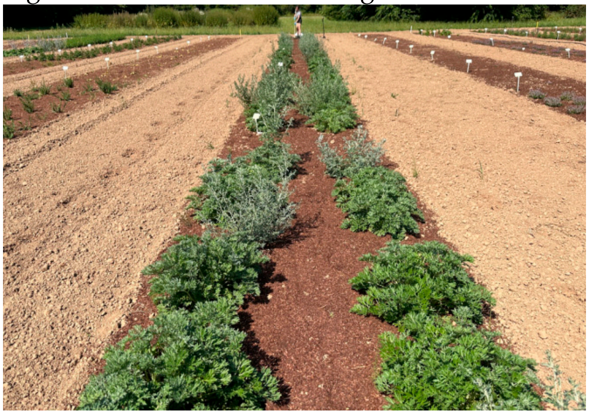

Figure S11. Plants in the first year of the vegetation

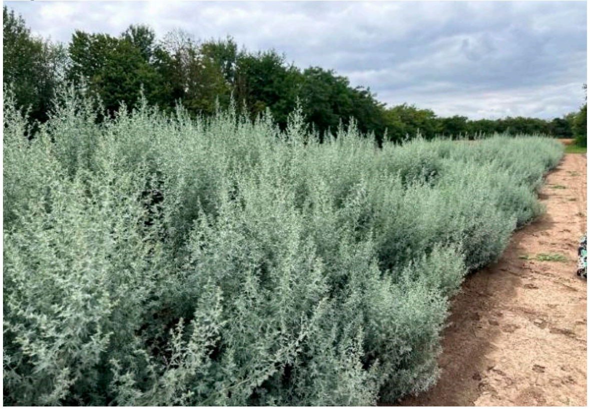

Figure S12. Plants in the second year of the vegetation

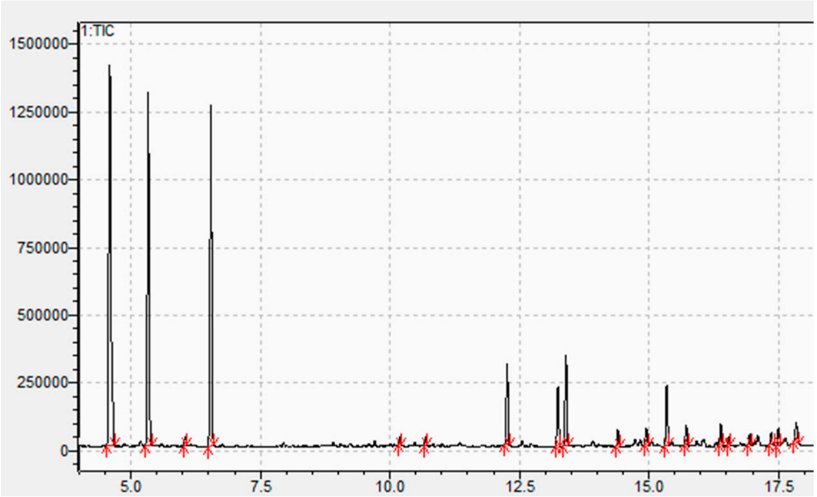

Figure S13. EO chromatogram of population 4

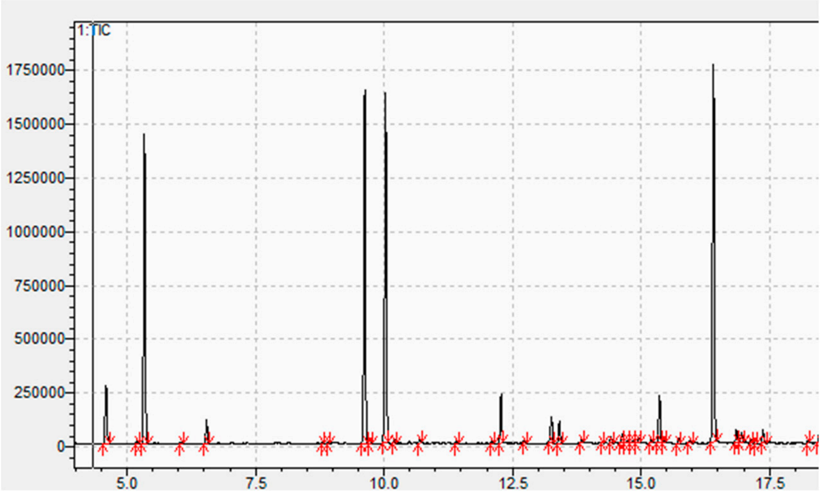

Figure S14. EO chromatogram of population 9

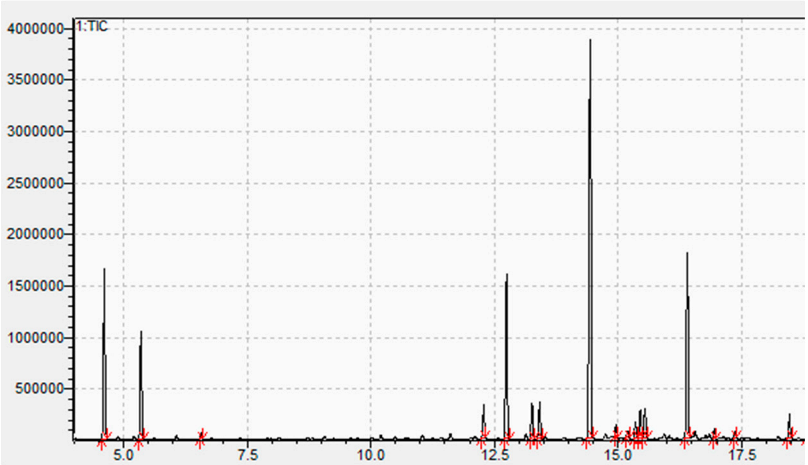

Figure S15. EO chromatogram of population 10

Table S1. One-way Analysis of Variance parameters (DF - the degrees of freedom; SS - the sum of squares; MS - the mean sum of squares; F -the F-statistic; P - the P-value).

		DF	SS	MS	F	P
Essential Oil	Population	10	2.22350	0.22235	24.184	0.000000
	Error	22	0.20227	0.00919		
	Total	32	2.42576			
Polyphenols	Population	10	0.16922	0.01692	11.379	0.000001
	Error	22	0.03272	0.00149		
	Total	32	0.20194			
Tannins	Population	10	0.072628	0.007263	6.986	0.000076
	Error	22	0.022872	0.001040		
	Total	32	0.095500			
Phenolic acids	Population	10	0.076105	0.007610	4.455	0.000528
	Error	33	0.056375	0.001708		
	Total	43	0.132480			
Plant height (cm)	Population	10	5668	567	2.756	0.009953
	Error	44	9050	206		
	Total	54	14718			
Number of shoots per plant	Population	10	853.0	85.3	0.3139	0.973445
	Error	44	11956.4	271.7		
	Total	54	12809.4			
Lenght of internodes (mm)	Population	10	314.40	31.44	2.492	0.035639
	Error	22	277.57	12.62		
	Total	32	591.98			
Lenght of inflorescences (cm)	Population	10	4587.8	458.8	2.275	0.051947
	Error	22	4436.9	201.7		
	Total	32	9024.7			
Dry herb	Population	10	1.87182	0.18718	1.2613	0.309585
	Error	44	3.26500	0.14841		
	Total	54	5.13682			

Proposal of screening strategy for thujone-free or low-thujone Artemisia absinthium chemotypes

The screening of candidate populations would be performed using gas chromatography-mass spectrometry (GC-MS), which is a standard and reliable analytical technique for the precise quantification of volatile compounds, including both α- and β-thujone isomers, in plant essential oils.

The selection criteria for breeding and medicinal purposes would be directly based on the maximum thujone intake limits established by the European Medicines Agency (EMA) and other relevant regulatory authorities. According to the EMA public statement (EMA/HMPC/732886/2012) the safe limit of exposure is defined as 6 mg per day for adults.

In practical terms, populations will be classified as "thujone-free" if both α- and β-thujone are not detected in the essential oil, i.e., their concentrations are below the limit of quantification of the analytical method. For "low-thujone" types, the total thujone content in the dried herb, calculated according to the recommended daily dose. Only those populations with the thujone content below the established threshold will be selected for further breeding and medicinal applications. Populations exceeding this limit will be excluded from pharmaceutical usage but may still be considered for non-food uses, such as biopesticides, where thujone's bioactivity is desirable and human exposure is not a concern.

This approach would guarantee that the selection of *Artemisia absinthium* populations for breeding and medicinal use are aligned with current international safety standards for thujone content, providing a solid scientific and regulatory foundation for further research and application.
